# Human-AI collaboration for ultrasound diagnosis of thyroid nodules: a clinical trial

**DOI:** 10.1007/s00405-025-09236-9

**Published:** 2025-02-08

**Authors:** Axel Bukhave Edström, Fatemeh Makouei, Kasper Wennervaldt, Anne Fog Lomholt, Mikkel Kaltoft, Jacob Melchiors, Gitte Bjørn Hvilsom, Magne Bech, Martin Tolsgaard, Tobias Todsen

**Affiliations:** 1 Department of Otorhinolaryngology, Head and Neck Surgery and Audiology, Rigshospitalet University Hospital of Copenhagen, 2100 Copenhagen, Denmark; 2 Institute of Clinical Medicine, Faculty of Health and Medical Sciences, University of Copenhagen, Blegdamsvej 3, 2200 Copenhagen, Denmark; 3 Department of Otorhinolaryngology, Head and Neck Surgery, Zealand University Hospital, 4600 Køge, Denmark; 4 Copenhagen Academy of Medical Education and Simulation (CAMES), Rigshospitalet University Hospital of Copenhagen, 2100 Copenhagen, Denmark; 5 Department of Obstetrics, Juliane Marie Centre, Rigshospitalet University Hospital of Copenhagen, 2100 Copenhagen, Denmark

**Keywords:** Thyroid lesions, Thyroid cancer, Diagnostic ultrasound, Artificial intelligence, Computer-assisted diagnosis (CAD)

## Abstract

**Purpose:**

This clinical trial examined how the articifial intelligence (AI)-based diagnostics system S-Detect for Thyroid influences the ultrasound diagnostic work-up of thyroid ultrasound (US) performed by different US users in clinical practice and how different US users influences the diagnostic accuracy of S-Detect.

**Methods:**

We conducted a clinical trial with 20 participants, including medical students, US novice physicians, and US experienced physicians. Five patients with thyroid nodules (one malignant and four benign) volunteered to undergo a thyroid US scan performed by all 20 participants using the same US systems with S-Detect installed. Participants performed a focused thyroid US on each patient case and made a nodule classification according to the European Thyroid Imaging Reporting And Data System (EU-TIRADS). They then performed a S-Detect analysis of the same nodule and were asked to re-evaluate their EU-TIRADS reporting. From the EU-TIRADS assessments by participants, we derived a biopsy recommendation outcome of whether fine needle aspiration biopsy (FNAB) was recommended.

**Results:**

The mean diagnostic accuracy for S-Detect was 71.3% (range 40–100%) among all participants, with no significant difference between the groups (p = 0.31). The accuracy of our biopsy recommendation outcome was 69.8% before and 69.2% after AI for all participants (p = 0.75).

**Conclusion:**

In this trial, we did not find S-Detect to improve the thyroid diagnostic work-up in clinical practice among novice and intermediate ultrasound operators. However, the operator had a substantial impact on the AI-generated ultrasound diagnosis, with a variation in diagnostic accuracy from 40 to 100%, despite the same patients and ultrasound machines being used in the trial.

## Introduction

Thyroid nodules are common in the general population with prevalence estimations of up to 67% [1]. The vast majority are benign, so it is crucial to carefully select the few needing further diagnostic work-up [1]. Ultrasound (US) is the first choice of medical imaging modality for assessing thyroid nodules and is used to decide whether to perform fine needle aspiration biopsy (FNAB) [2, 3]. The Thyroid Imaging Reporting And Data System (TIRADS) is a classification system used to standardise the risk assessment of malignancy in thyroid nodules and to guide whether the clinician should perform a FNAB [4]. However, US is an operator-dependent imaging modality [5], and there is considerable variability in the interpretation of thyroid nodule sonographic features [6]. Therefore, artificial intelligence(AI)-based systems have been developed for US machines to decrease user variability [7].

S-Detect for Thyroid (Samsung Medison, Co., Ltd., Seoul, Korea) is the first deep learning-based program to automatically measure size, describe the TIRADS characteristics, and diagnose thyroid nodules [8]. Several studies have found that S-Detect can improve junior physicians’ accuracy in predicting thyroid nodule malignancy when interpreting US images [9–14]. In clinical practice however, the US exam is not used to diagnose whether a nodule is malignant but to assess the risk of malignancy with a TIRADS score and decide whether an FNAB should be performed. Furthermore, previous studies used very experienced operators to perform the thyroid US exam and selected the best image for AI analysis [10–14]. However, the quality of the stored US images is also operator-dependent, and it is unknown how S-Detect will change the diagnostic work-up of thyroid nodules when incorporated in the US examination by operators of different experience levels.

The aims of this clinical trial were twofold: firstly, to explore how S-Detect would affect thyroid nodule assessment for different US operators, and secondly, how the US operator experience influences the diagnostic accuracy of S-Detect.

## Methods

We conducted a prospective clinical trial at the Department of Otorhinolaryngology, Head and Neck Surgery and Audiology, Rigshospitalet, Copenhagen Denmark, where 20 physicians and medical students conducted thyroid US exams including S-Detect evaluation of the same five patients with thyroid nodules.

The participants were invited to represent three different head and neck US experience levels from novice to intermediate US users: (I) last year medical students (student group), (II) first-year ENT registrars (US novice group), and (III) senior ENT registrars using US on a weekly basis (US experienced group). The ‘student group’ were last year medical students from the University of Copenhagen with no prior experience with thyroid US, but who, prior to the trial, had completed a day of hands-on training in point-of-care abdominal and musculoskeletal US as part of the curriculum at the medical school. As part of the trial, the student participants received two hours of formal thyroid US training before scanning the patients in the trial. This training included a one-hour lecture on basic thyroid US and the European TIRADS (EU-TIRADS) system [15], followed by an hour of supervised hands-on thyroid US practice on each other in pairs.

In Denmark, head and neck US is an integrated part of the clinical ENT examination, and the patients seen in the ENT department are not referred to the radiology department, but instead have an US performed by an ENT surgeon if needed. Therefore, formal head and neck US training is provided through the second year of the Otorhinolaryngology, Head and Neck Surgery Specialist Training in Denmark. As such, the 1st year registrars in the ‘US novice group’ in this trial were expected to perform supervised head and neck US examinations in the clinic. The criteria for the ‘US experienced group’ were senior ENT registrar who had formal US training, performed > 100 neck US exams, and independently used US weekly in the outpatient clinic.

For the five patients recruited for the trial, inclusion criteria were presence of a thyroid nodule that had been referred to the ENT department and a cytology report on the nodule from a FNAB. Exclusion criteria was any previous thyroid surgery.

**Fig. 1:**
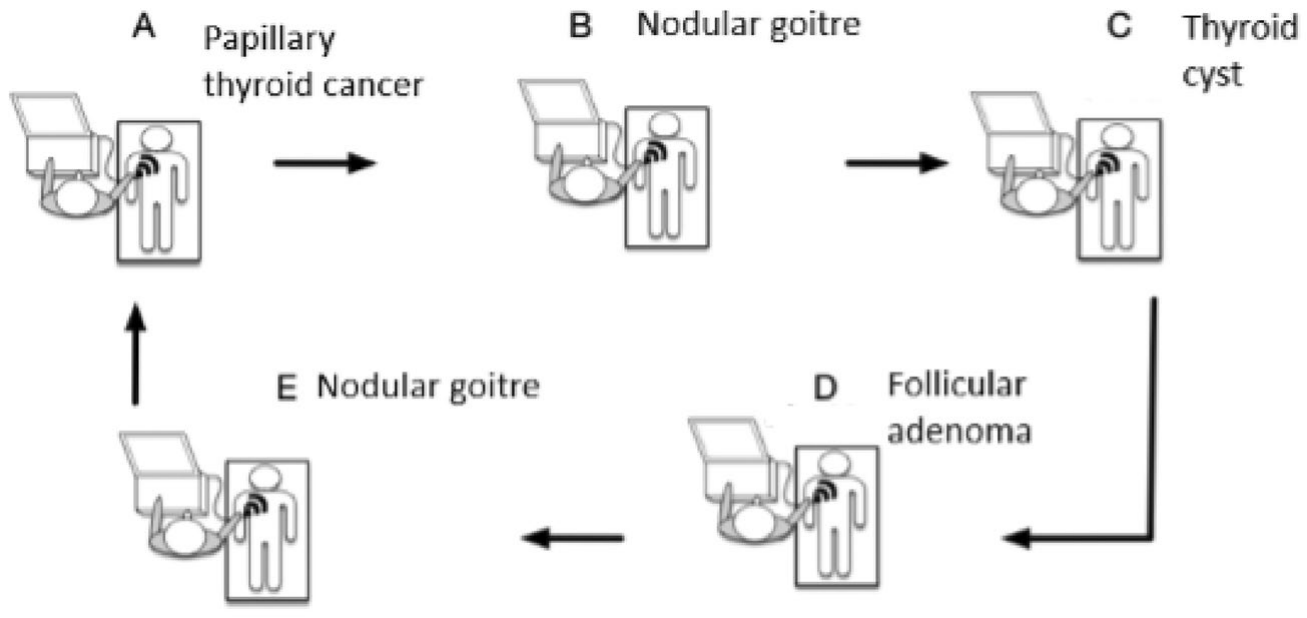
Illustration of the experimental setup with patient diagnoses displayed. Five patients each with a thyroid nodule placed on one of five stations (**A**–**E**) each with an ultrasound system with S-Detect for Thyroid installed. Five participants each had 10 min to scan the patients, use the program, and record their findings before switching to the next station in the loop. S-Detect for Thyroid = Artificial intelligence-based program for diagnosing thyroid nodules

The participants in the three groups performed thyroid US on the same patient cases in an experimental setup. The setup consisted of five stations (A-E), each with an US system with S-Detect installed and a patient with a thyroid nodule (Fig. 1). The US equipment used in the study included two Samsung V7, two Samsung V8, and a Samsung RS85 Prestige, all with high-frequency linear array probes of either 2–14 or 4–18 MHz (see Appendix A for details). All machines had the real-time AI-based diagnostic system S-Detect for Thyroid installed, with the program set to the default “High Accuracy” setting and used EU-TIRADS for classification of the thyroid nodules.

At each station, participants were instructed to perform a systematic thyroid scan on the patient, measure the nodule, and assess it using EU-TIRADS classification. Afterward, they chose the best frame to perform an S-Detect evaluation on the US machine. The participants recorded their own EU-TIRADS nodule assessments and the S-Detect diagnosis on paper forms that were provided. Finally, they were asked to assess the nodule with EU-TIRADS a second time after receiving input from the S-Detect diagnosis and document this assessment. The participants had 10 min at each station to perform a thyroid US examination and document their own and S-Detect's thyroid nodule assessment before they moved to the next station in an Objective Structured Clinical Examination (OSCE)-like test setting [16]. A set of instructions for the procedure was present at every station, along with a short case description unique to each patient, designed to guide participants to assess the same thyroid nodules (see Appendix B). Participants were blinded to previous in-clinic nodule assessments and cytology reports of the patients’ nodules.

Two head and neck US experts (senior consultants in head and neck surgery highly experienced in US and faculty on international head and neck US courses) assessed participants’ US skills using the validated Objective Structured Assessment of Ultrasound Skills (OSAUS) tool [17] (score range 1–5). The participants received their OSAUS scores based on their US performance on two stations, station A and station D, and a mean score from the two assessors was calculated.

After the participants completed the US scans, they filled out a survey regarding their experience with head and neck US, as well as their views on AI in US diagnostics (see Appendix B).

To validate the US diagnosis, three consultants in head and neck surgery, with expertise in neck US, scanned all the patients’ cases and discussed the EU-TIRADS nodule classifications and measurements until they agreed on a consensus for the gold standard.

### S-Detect

**Fig. 2:**
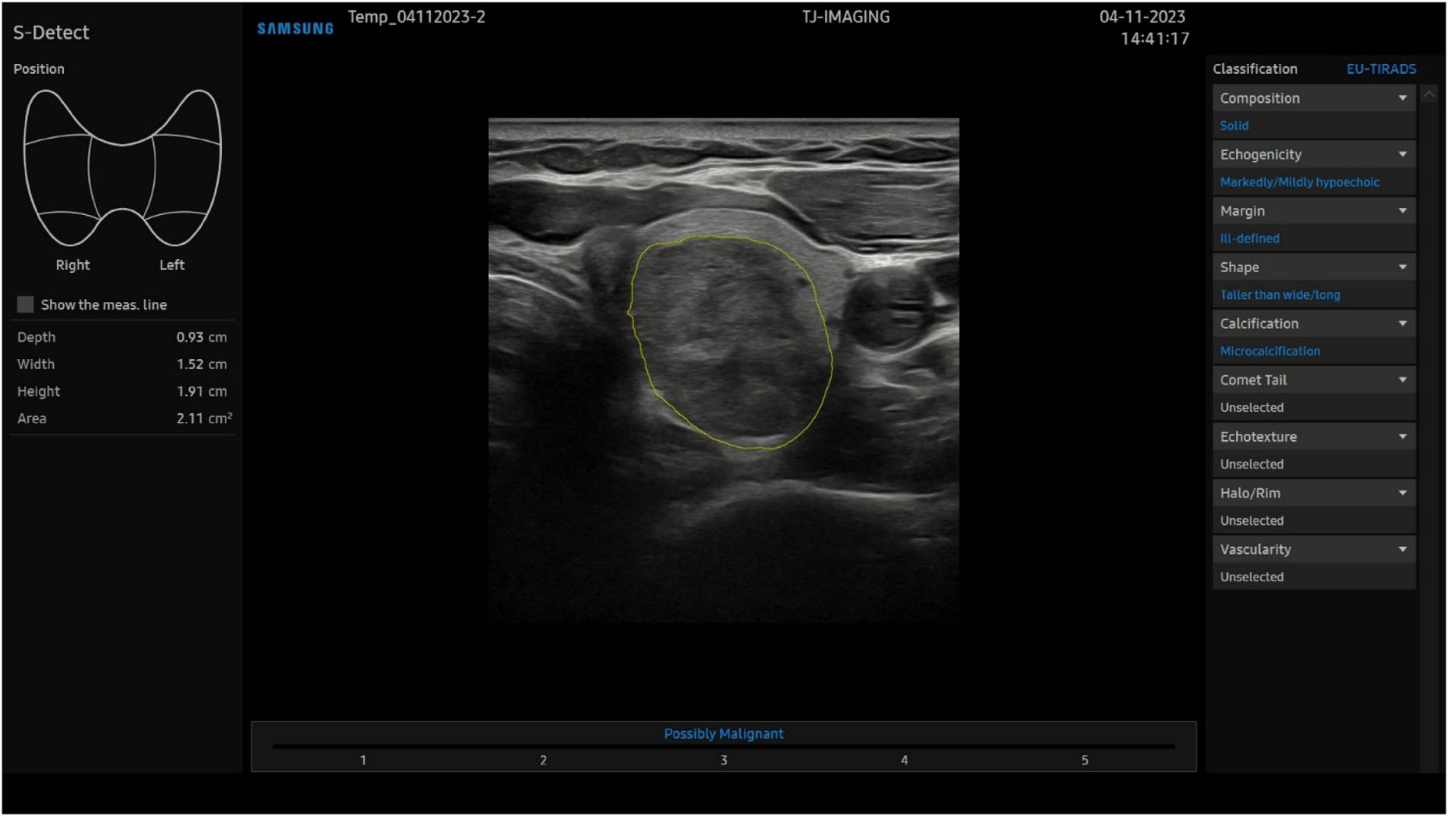
Greyscale US transverse image of a left thyroid lobe with S-Detect’s analysis of a thyroid nodule. Nodule measurements are on the left, nodule classification in EU-TIRADS on the right, and the diagnosis as “Possibly Malignant” on the bottom. US = Ultrasound. S-Detect = Artificial intelligence-based program for diagnosing thyroid nodules. EU-TIRADS = European Thyroid Imaging Reporting And Data System

The participants in all three groups received a 30-min training session in using S-Detect for Thyroid before scanning the patients in the trial. After completing the thyroid US examination in the trial, the participants froze a transverse B-mode image that best represented the thyroid nodule for S-Detect evaluation. They then selected the region of interest including the thyroid nodule, with contours drawn either automatically, with S-Detect, or manually. S-Detect then measured the nodule, gave an EU-TIRADS nodule description, and diagnosed the nodule as either “Possibly benign” or “Possibly malignant”. Since S-Detect was only used in the transverse plane in our experiment, no cranial-caudal length measurements were done by the system, only transverse height and width. See Fig. 2 for an example of a S-Detect evaluation.

### Outcomes

This study had two primary outcomes:

- “S-Detect diagnostic accuracy”—S-Detect nodule diagnosis as either benign or malignant with cytology/histology report as the gold standard.
- “Biopsy recommendation”—FNAB recommendation by participants before and after AI assistance with expert assessment as the gold standard.

Secondary outcomes were the OSAUS scores of participants and nodule measurements by participants and AI.

For the S-Detect diagnostic accuracy outcome, the postoperative histology report was the gold standard. If histology was not available, the preoperative cytology report was the gold standard. From the EU-TIRADS score and measurements of a nodule as assessed by the participants, the biopsy recommendation outcome was derived from each nodule analysis, both before and after AI assistance (see Table 1). The same derivation was applied to each patient’s expert consensus assessment to be used as the gold standard. Biopsy recommendation was not derived from S-Detect assessments, as the program does not distinguish between mildly hypoechoic and markedly hypoechoic in its assessment of thyroid nodules, making it impossible to distinguish between EU-TIRADS 4 and 5 nodules.

**Table 1:** EU-TIRADS criteria for recommendation of FNAB of thyroid nodules

	EU-TIRADS 2	EU-TIRADS 3	EU-TIRADS 4	EU-TIRADS 5
FNAB recommended when:	No FNAB recommended	Nodule size > 20 mm	Nodule size > 15 mm	Nodule size > 10 mm

*EUTIRADS* European Thyroid Imaging Reporting And Data System, *FNAB* Fine needle aspiration biopsy.

### Statistics

The Kruskal–Wallis test was used to test for differences in S-Detect diagnosis and biopsy recommendation accuracy both before and after AI assistance, between the three participant groups. We used the Wilcoxon test to compare the participants’ results in biopsy recommendation before and after AI assistance.

To compare the participants’ and S-Detect nodule size measurements, we used the mean difference from the expert measurements in mm and compared it with a two-sample t-test. We also compared the OSAUS scores between groups using the two-sample t-test.

## Results

### Patient cases

All five recruited patients were female. We recruited one patient with an EU-TIRADS 2 cystic nodule diagnosed as a benign (Bethesda II) on the preoperative cytology report, one with an EU-TIRADS 3 nodular goitre diagnosed as benign (Bethesda II) on preoperative cytology, and three with EU-TIRADS 5. One of the EU-TIRADS 5 nodules was confirmed as papillary thyroid cancer on the postoperative histology report, while another was diagnosed as follicular adenoma on the postoperative histology report. The last EU-TIRADS 5 nodule was nodular goitre diagnosed as benign (Bethesda II) on preoperative cytology. The patients’ nodules are described in Table 2.

**Table 2:** Summary of patients and their thyroid nodules’ characteristics for the study experiment

	Patient A	Patient B	Patient C	Patient D	Patient E
EU-TIRADS	5	3	2	5	5
FNAB recommendation	Yes	Yes	No	Yes	Yes
Cytology/histology of nodule	Papillary Thyroid Cancer	Nodular goitre	Thyroid cyst	Follicular adenoma	Nodular goitre
Nodule height	7 mm	17 mm	23 mm	30 mm	17 mm
Nodule width	13 mm	28 mm	25 mm	29 mm	14 mm
Nodule length	17 mm	> 40 mm	31 mm	35 mm	27 mm
Composition	Solid	Mixed solid/cystic	Cystic	Solid	Solid
Echogenicity	Markedly hypoechoic	Isoechoic	Anechoic	Isoechoic	Mildly hypoechoic
Margin	Smooth	Smooth	Smooth	Irregular/ill-defined	Irregular/ill-defined
Shape	Round/oval	Round/oval	Round/oval	Taller than wide	Taller than wide
Calcification	None	Macrocalcification	None	Microcalcification	Microcalcification

Nodule measurements, US characteristics, EU-TIRADS scores, and FNAB suggestions are from consensus assessments by three head-and-neck US experts. The dimensions of height, width, and length refer to the nodules’ transversal height, transversal width, and longitudinal width respectively

*EU-TIRADS* European Thyroid Imaging Reporting And Data System, *FNAB* Fine needle aspiration biopsy, *US* Ultrasound

### Participant groups

We recruited 20 participants for the study: eight last-year medical students (student group), three junior ENT registrars (US novice group), and nine senior ENT registrars (US experienced group). None of the medical students had prior head and neck US experience, while the junior ENT registrars had performed a mean of 35 (range 0–70) independent head and neck US scans.

The senior ENT registrars all had formal head and neck US training, performed a mean of 461 (range 100–1000) independent head and neck US scans, and 4.3 (range 2–7) mean years of US experience (see Table 3 for demographics).

**Table 3:** Participant group size and US experience. Ranges are shown in brackets

	Student group	US novice group	US experienced group
No. of individuals	8	3	9
Male	50%	100%	56%
Mean OSAUS score	2.6 (1.7–3.1)	2.7 (2.3–3.4)	4.3 (4.0–4.5)
Mean years of US experience	0	1.3 (1–2)	4.5 (3–7)
Mean estimated no. of independent HNUS scans performed	0	35 (0–75)	461 (100–1000)
Previously attended HNUS course(s)	0%	33%	100%
Mean no. of previous HNUS courses (if any attended)	–	1	2.2 (1–4)
How often US is used in participant’s work	Never or less than monthly	Weekly-monthly	Daily-weekly

*US* Ultrasound, *HNUS* Head-and-neck ultrasound, *OSAUS* Objective structured assessment of ultrasound skills, ranges from 1 to 5

From the 20 participants and 5 cases, we had 100 analysed cases in our trial. However, there was missing data. No S-Detect diagnosis was recorded in two patient cases, leaving 98 analysed cases for this outcome. In 10 patient cases, the biopsy recommendation could not be derived from assessments by the participant, leaving 90 analysed cases for this outcome.

The mean OSAUS score (evaluating their US performance) was 2.6 (SD 0.52), 2.7 (SD 0.61), and 4.3 (SD 0.19) for the student, US novice, and US experienced groups respectively. No significant difference was observed between OSAUS scores for the student and US novice groups (p = 0.72), while significant differences were observed between both the student and US novice groups when compared to the US experienced group (p < 0.01 and p = 0.046 respectively).

### Accuracy outcomes

The mean diagnostic accuracy using S-Detect was 71.3% (range 40–100%), in predicting whether a nodule was benign or malignant. The mean S-Detect diagnostic accuracy was 74.4% in the student group, 60% in the US novice group, and 72.2% in the US experienced group with no significant difference between the groups (p = 0.31).

Biopsy recommendation accuracy for all groups compiled before and after AI were 69.8% and 69.3% (p = 0.75) respectively. Biopsy recommendation accuracy was 74.4% before AI assistance and 67.1% after AI assistance in the student group (p = 0.33), 60% before AI and 71.7% after AI in the US novice group (p = 0.37), and 68.9% before AI and 70.4% after AI in the US experienced group (p = 1). No statistical significance was observed when comparing biopsy recommendation results between the groups, both for results before and after AI assistance (p = 0.47 and p = 0.94 respectively). See Table 4 for all accuracy outcomes.

**Table 4:** Results of assessments by AI alone and participants before and after AI assistance, stratified in three participant groups

	Student group	US novice group	US experienced group	All participants
AI diagnostic accuracy	74.4% (60–100%)	60% (60–60%)	72.2% (40–100%)	71.3% (40–100%)
Participant biopsy recommendation accuracy				
Before AI	74.4% (60–100%)	60% (40–80%)	68.9% (60–80%)	69.8% (40–100%)
After AI	67.1% (40–100%)	71.7% (60–80%)	70.4% (33–100%)	69.3% (33–100%)

The AI diagnosis is of benign/malignant status of thyroid nodules with cytology/histology report as the gold standard. Biopsy recommendation (before and after AI assistance) is a decision on whether to do a biopsy of thyroid nodules based on ultrasound assessment with expert assessment as the gold standard. Ranges are shown in brackets

*EU-TIRADS*  European Thyroid Imaging Reporting And Data System. *AI* = Artificial intelligence

**Fig. 3:**
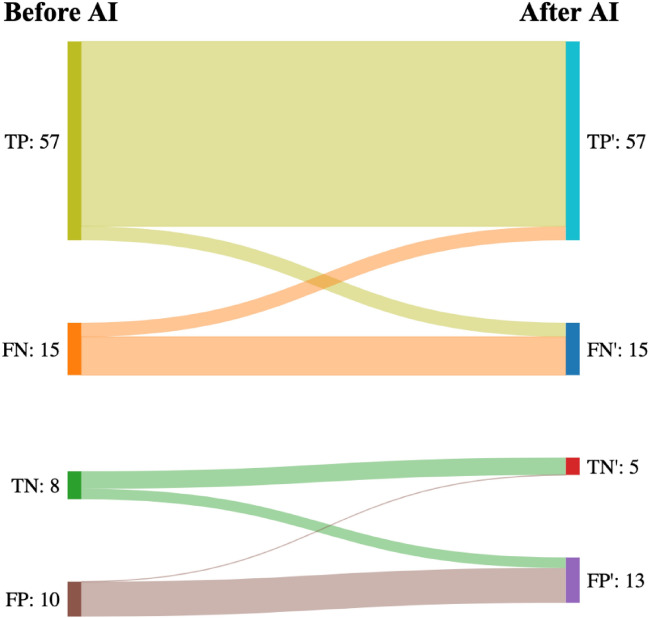
Sankey diagram of changes in biopsy recommendation by participants before and after AI assistance. Left-hand side is before AI, right-hand side (marked ‘) is after AI. Biopsy recommendation is a decision on whether to do a biopsy of thyroid nodules based on ultrasound assessment with expert assessment as the gold standard. *TP* True positive, *TN* True negative, *FP* False positive, *FN* False negative, *AI* Artificial intelligence

In 11 (12%) cases, participants changed their biopsy recommendation due to feedback from S-Detect. Of these, four changed from an incorrect to a correct biopsy recommendation, while seven changed from a correct to an incorrect biopsy recommendation (see Fig. 3). Of the 11 changed cases, seven were done by student participants, three by US novice participants, one by an US experienced participant. All five patient cases were represented in the 11 changed cases at least once, with patient case B occurring the most (four times). See Appendix C, Table 6 for an overview of these cases.

S-Detect diagnostic accuracy for identifying the malignant nodule in patient A was 15%, for the thyroid cyst and the follicular adenoma in patients C and D 100%, and for the nodular goitre lesions of patients B and E 90% and 55% respectively. For detailed results stratified in the five patient cases, see Appendix C, Table 7.

### Nodule measurements

When measuring the height of nodules, participants achieved a mean difference from the gold standard of 1.9 mm (SD 2.3) with S-Detect achieving a difference of 1.8 mm (SD 2.2) (p = 0.85). For measurement of nodule width, participants’ mean difference was 1.7 mm (SD 1.7), while S-Detect's mean difference was 2.4 mm (SD 1.8) (p = 0.01). For mean differences of measurement for each patient, see Appendix C, Table 8.

## Discussion

In this clinical trial, we observed that using S-Detect did not improve the thyroid diagnostic work-up of thyroid nodules among novice and intermediate US operators. Furthermore, we observed that the operator had a strong impact on the AI-generated US diagnosis, with a variation in diagnostic accuracy ranging from 40 to 100%, even though the same patients and US machines were used in the trial.

A strength of our study is the controlled experimental setup, including participants with different US experiences who used S-Detect on the same US equipment and patient cases. In this way, we could measure the impact of the US operator’s influence on the diagnostic accuracy of AI-assisted thyroid nodule diagnosis and how AI influenced the operator’s assessments. Another strength is that we had a cytological/histological diagnosis on all the patients, and three experts were used to define the reference standard for the US characteristics.

There were also limitations to this study. We only had five patient cases (four benign and one with thyroid cancer) who volunteered to have additional thyroid US exams performed. These few cases can, therefore, not be expected to represent the general population referred for diagnostic work-up. Another limitation was the uneven group sizes in our study. Our US novice group consisted of only three individuals, while the other groups consisted of eight and nine, respectively. This decreased statistical power and made detecting a true difference between US novices and the other groups harder. However, 20 different US operators analysed the cases, and a total of 100 US cases clustered within the five patient cases that were analysed in the study. Thus, this study design allowed us to measure the US operator’s variability for S-Detect diagnostic accuracy.

To the best of our knowledge, this is the first study to directly compare the variance in diagnostic accuracy of S-Detect for thyroid nodule evaluation of the same patients but with different US operators. Previous research used images obtained by experienced operators for S-Detect analysis and compared the diagnostic accuracy with physicians of different US experience levels who retrospectively analysed the same images [11, 13, 18, 19]. These studies did not involve different novice operators conducting the US examinations and acquiring images independently for S-Detect’s analysis. Our study design with various US operators collecting images for S-Detect analysis is closer to clinical practice. This might explain why we did not find S-Detect to improve the diagnostic accuracy of novice US operators compared to previous studies.

The difference in diagnostic outcomes between our study and previous studies might explain why assistance from S-Detect did not enhance diagnostic accuracy. S-Detect will not strictly provide a biopsy recommendation but rather a “possibly benign” or “possibly malignant” diagnosis, and the physician has therefore been forced to make the same binary diagnosis to compare with the diagnostic accuracy of S-Detect in previous studies [10, 12]. However, US cannot make malignant diagnoses in the diagnostic work-up of thyroid nodules in a clinical setting [20]. Still, it can evaluate malignancy risk and recommend a FNAB to be performed. Therefore, we converted the nodule classifications and measurements to a biopsy recommendation based on EU-TIRADS classification to measure the effect on real-world clinical decision-making.

We observed a variation between 40 and 100% in diagnostic accuracy from S-Detect depending on the US operator when the same US equipment and patients were examined. This may be best explained by operator-dependent variation in the US images used for S-Detect analyses. Since the system can only do analyses on two-dimensional sections of nodules, different operators’ choices of what nodule sections to present to the program can mean the difference between a benign and malignant diagnosis by S-Detect, even on the same nodule. This has previously not been tested on thyroid nodules. Still, previous studies investigating S-Detect for Breast found an inconsistency when the AI diagnosed lesions in two different planes in 11.6–18.1% of the cases [21, 22].

Although previous studies have reported better diagnostic accuracy of S-Detect when compared to novice physicians [11–14], our trial suggests that it may not directly translate to improved diagnostic work-up in clinical practice. Unlike static imaging modalities like X-ray and cross-sectional imaging, these findings may be attributed to the operator-dependent quality of US images. This raises concerns about the feasibility of applying deep learning to interpret US images, as less-skilled operators could provide suboptimal input data to the AI system. We believe that for AI-assisted US diagnosis to be successfully implemented in clinical settings, the technology should be developed with a clear purpose as a tool for the clinician in mind. Future research should focus more on how AI-assisted tools are affected by the image quality obtained by the US operator and how they affect the diagnostic process, including time use and patient outcomes.

In conclusion, we observed that using S-Detect did not enhance the diagnostic accuracy of thyroid nodule evaluation among novice and intermediate US operators. The operator significantly influenced the AI-generated US diagnosis, with diagnostic accuracy ranging from 40 to 100%, despite the same patients and US machines used. While AI systems like S-Detect have the potential to improve the diagnostic work-up of thyroid nodules, more research is needed to ensure how it should be used in the diagnostic process to save time and improve final patient outcomes.

## Data Availability

The data that support the findings of this study are available from the corresponding author, A. B. Edström, upon reasonable request.

## Appendix A

See Table 5.

**Table 5:** Overview of ultrasound equipment placed on each of the experimental setup’s five stations

	Station A	Station B	Station C	Station D	Station E
Ultrasound equipment	Samsung V7 with linear array probe 2–14 MHz	Samsung V7 with linear array probe 2–14 MHz	Samsung V8 with linear array probe 2–14 MHz	Samsung RS85 Prestige with linear array probe 2–14 MHz	Samsung V8 with linear array probe 4–18 MHz

## Appendix B

The EU-TIRADS paper forms used by participants to record their own and S-Detect’s analyses included nodule measurements of height, width, and length, as well as classification of the nodule in the lexicons composition (solid, cystic, mixed solid/cystic, or spongiform appearance), echogenicity (hyperechoic, isoechoic, mildly hypoechoic, or markedly hypoechoic), margin (smooth, irregular, or ill-defined), shape (oval, round, or taller-than-wide), and calcification (none, macrocalcification, or microcalcification). The forms also required participants to answer with an EUTIRADS score before and after using S-Detect and to record S-Detect’s binary diagnosis.

The case descriptions for each patient’s station were as follows:

- Station A case: Hypofunctioning area in right thyroid lobe found on scintigraphy. No symptoms related to pressure, hypo-, or hyperthyroidism.
- Station B case: Pressure-related symptoms on right side of neck radiating towards ear for a while.
- Station C case: Pain in right side of neck, now noticed swelling in the area. Feels voice has become hoarser.
- Station D case: Dry cough for years, feels it has worsened. Scintigraphy reveals cold nodule in left thyroid lobe.
- Station E case: Increased FDG-uptake in left thyroid lobe found on PET-CT in relation to another medical evaluation.

The OSAUS assessments included four points: applied knowledge of ultrasound equipment, image optimization, systematic examination, and interpretation of images.

The questionnaire contained questions on ultrasound experience: “How many years of ultrasound experience do you have?”, “How many head-and-neck ultrasound scans have you independently performed? (roughly)”, “Have you previously attended a head-and-neck ultrasound course?”, “If yes, how many?”, and “How often do you use ultrasound scan in your work? Daily, weekly, monthly, or never/less than monthly?”.

## Appendix C

See Tables 6, 7 and 8.

**Table 6:** Overview of changes in biopsy recommendation by participants after AI assistance

Type of change in diagnosis	Participant group	Patient case
TP to FN	Student	A
TP to FN	Student	B
TP to FN	Student	D
TP to FN	US novice	A
TN to FP	Student	C
TN to FP	Student	C
TN to FP	US experienced	C
FN to TP	Student	B
FN to TP	Student	E
FN to TP	US novice	B
FN to TP	US novice	B

Biopsy recommendation is a decision on whether to do biopsy of thyroid nodules based on US assessment with expert assessment as the gold standard. The patient cases (A-E) are five patients each with a thyroid nodule assessed by the participants

*AI* Artificial intelligence, *US* Ultrasound, *TP* True positive, *FP* False positive, *TN* True negative, *FN* False negative

**Table 7:** Results of assessments by AI alone and participants before and after AI assistance, stratified in the five patient cases

	Cytology/histology of nodule	Patient A	Patient B	Patient C	Patient D	Patient E
		Papillary thyroid cancer	Nodular goitre (benign)	Thyroid cyst (benign)	Follicular adenoma (benign)	Nodular goitre (benign)
AI diagnosis	Accuracy	15%	90%	100%	100%	55%
Participant assessment (before AI)	Biopsy recommendation accuracy	67%	70%	40%	85%	85%
Participant assessment (after AI)	Biopsy recommendation accuracy	53%	75%	28%	80%	100%

AI diagnosis is an AI program’s benign/malignant diagnosis of thyroid nodule with cytology/histology report as the gold standard. Biopsy recommendation (before and after AI assistance) is a decision on whether to do biopsy of thyroid nodules based on US assessment with expert assessment as the gold standard. Ranges are shown in brackets

*US* Ultrasound, *AI* Artificial intelligence.

**Table 8:** Mean difference of thyroid nodule measurements by participants and AI

		Patient A	Patient B	Patient C	Patient D	Patient E	All patients
Nodule height mean difference	Participant	0.4 mm	2.2 mm	2.4 mm	3.1 mm	1.5 mm	1.9 mm
	AI	0.3 mm	3.4 mm	1.5 mm	2.4 mm	1.8 mm	1.8 mm
Nodule width mean difference	Participant	1.5 mm	1.4 mm	2.3 mm	1.6 mm	1.7 mm	1.7 mm
	AI	1.3 mm	1.8 mm	2.5 mm	3.0 mm	3.6 mm	2.4 mm

Height and width are from transversal ultrasound images of thyroid nodules

*AI* artificial intelligence
